# Recitation in Medical Colleges

**Published:** 1868-12

**Authors:** 


### EDITORIAL DEPARTMENT.

*Recitation in Medical Colleges.*—An editorial in the *Medical Record* strongly advocates the necessity of recitations in medical colleges, and unfavorably compares the graduates of recent times, with those "who flourished two centuries ago, and who obtained their knowledge by pounding drugs in the office of the village doctor, reading his books while he was away from home, and reciting their contents during the hours of leisure;" asserting that these latter were as thoroughly acquainted with the principles of their art as any men the world has ever seen.

The *Record*, however, does not advocate the abandonment of instructions by means of lectures, but contends that the session each year should be lengthened and the number of lectures diminished.

This plan would answer very well if the sessions were of the same length as those of literary institutions, extending throughout the greater part of the year. But such a lengthening of the sessions would, owing to the increased expense, prevent a great many students from attending and therefore seriously injure the colleges.

As shorter sessions therefore, are necessary, the plan of thorough weekly examinations would go far towards compensating for the want of recitations. In the *Baltimore College of Dental Surgery* at the close of each week, regular examinations are held on the lectures delivered during the week; not only for the purpose of determining the studious habits of each student, but as a review of these lectures.

In medical colleges the large number of students render these examinations of very little account; but the smaller number of students in dental colleges enable the Professors to devote considerable time to each student, thereby making these examinations something more than "meager quizzes."

